# Exploiting the Redox Activity of MIL-100(Fe) Carrier Enables Prolonged Carvacrol Antimicrobial Activity

**DOI:** 10.1021/acsami.1c21555

**Published:** 2022-02-18

**Authors:** Katia Caamaño, Raquel Heras-Mozos, Joaquín Calbo, Jesús Cases Díaz, João C. Waerenborgh, Bruno J. C. Vieira, Pilar Hernández-Muñoz, Rafael Gavara, Mónica Giménez-Marqués

**Affiliations:** † Instituto de Ciencia Molecular (ICMol), Universidad de Valencia, c/Catedrático José Beltrán 2, 46980 Paterna, Spain; ‡ Instituto de Agroquímica y Tecnología de Alimentos, IATA-CSIC, Av. Agustín Escardino 7, 46980 Paterna, Spain; § C2TN, DECN, Instituto Superior Técnico, Universidade de Lisboa, EN10, P-2695-066 Bobadela LRS, Portugal

**Keywords:** MOFs, controlled delivery, biocomposites, antimicrobial activity, food packaging

## Abstract

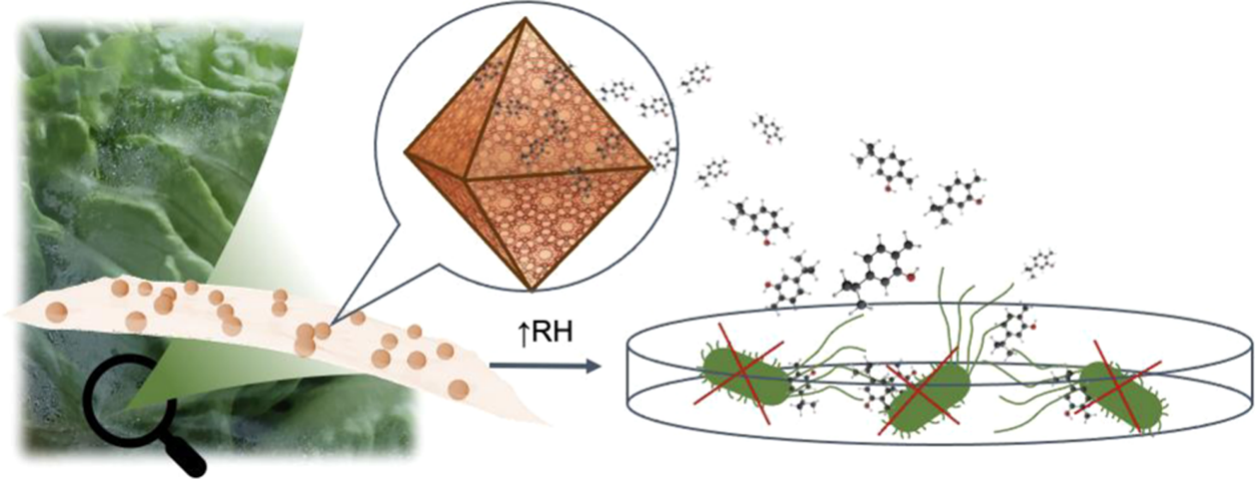

The design of efficient food contact materials that maintain optimal levels of food safety is of paramount relevance to reduce the increasing number of foodborne illnesses. In this work, we develop a smart composite metal–organic framework (MOF)-based material that fosters a unique prolonged antibacterial activity. The composite is obtained by entrapping a natural food preserving molecule, carvacrol, into a mesoporous MIL-100(Fe) material following a direct and biocompatible impregnation method, and obtaining particularly high payloads. By exploiting the intrinsic redox nature of the MIL-100(Fe) material, it is possible to achieve a prolonged activity against *Escherichia coli* and *Listeria innocua* due to a triggered two-step carvacrol release from films containing the carvacrol@MOF composite. Essentially, it was discovered that based on the underlying chemical interaction between MIL-100(Fe) and carvacrol, it is possible to undergo a reversible charge-transfer process between the metallic MOF counterpart and carvacrol upon certain chemical stimuli. During this process, the preferred carvacrol binding site was monitored by infrared, Mössbauer, and electron paramagnetic resonance spectroscopies, and the results are supported by theoretical calculations.

## Introduction

Ensuring food quality and safety is a major global challenge in a society severely affected by foodborne diseases. In this context, naturally occurring bioactive compounds (BAC) (e.g., antioxidants, vitamins, polyphenols) are effectively used as food flavoring agents and/or preservatives to inhibit microbial growth.^1^ Among them, phenolic compounds have recently attracted attention due to their antioxidant and antitumor activities.^2,3^ Despite exhibiting these properties, a desired large-scale use of these compounds is hampered by their volatile and insoluble nature, their susceptibility to various environmental and processing conditions, and their characteristic strong aroma. For these reasons, the encapsulation of volatile preservatives into carrier materials has been widely investigated in the food industry and the biomedical field as a plausible solution to optimize their activity.^4,5^

Nanometric porous metal–organic frameworks (nanoMOFs) have recently emerged as a promising alternative to ensure the safety and quality of food owing to their excellent porosity, high loading capacity, controlled release ability, and ease of surface modification.^6,7^ In some cases, their biocompatibility, processability, and large-scale production have fulfilled industrial requirements and potential biorelated uses including drug delivery, bioimaging, biosensing, and antibacterial activity.^8−10^ Linked to their performance as encapsulating agents, MOFs have been recently evaluated in the field of food safety for the removal of contaminants from production sources,^11^ in food packaging,^12^ improving the preservation of food,^13,14^ and in the detection and monitoring of contaminants in food products,^15^ among others. As compared to classical carrier agents like nano- and microemulsions,^16−19^ lipid nanoparticles,^20,21^ or liposomes,^22,23^ MOFs are particularly interesting since not only provide a high drug loading but also offer a controlled release.^24,25^ This is due to the high and regular porosity found in the MOF structures as well as the multiple organic/inorganic groups available for interaction with the guests. As a result, it is possible to tune the uptake and delivery of guest molecules by appropriate selection of the MOF scaffold. This chemical control provides a potential way to design carrier materials for the programmed release of multiple active ingredients occurring upon certain physical (light, temperature) or chemical stimuli (humidity, pH, chelating agents).^26^ Particular examples in the field of food safety include the encapsulation of eugenol in a PUM168 single crystal^27^ or the triggered release of allyl isothiocyanate.^28,29^

Among the possible MOFs to be used as encapsulating agents in food-related applications, mesoporous iron(III) trimesate nanoMOF MIL-100(Fe) offers unique possibilities since it is biocompatible and can be produced through green synthesis^30^ in optimal scales.^31,32^ Essentially, the structure characteristics of MIL-100(Fe) permit to form accessible coordinatively unsaturated iron sites upon induced reducibility on the framework, strongly modifying the preferred interactions with guest molecules.^33^ Moreover, its feasible nanostructuration improves its colloidal stability, thus facilitating the processing of the material for immediate applications like its integration in films.^34^

**Figure 1:**
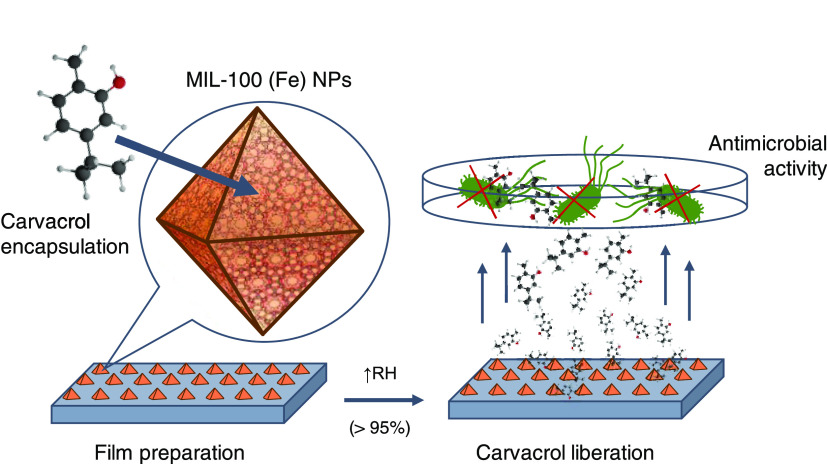
Schematic representation of the carvacrol encapsulation process into MIL-100(Fe) nanoparticles (NPs) and their implementation in polymeric films, showcasing the antimicrobial properties of the liberated carvacrol molecules upon exposure to high relative humidity (RH) simulating fresh food conditions.

In this work, a composite material based on a phenolic compound, carvacrol, loaded into nanoMIL-100(Fe) is obtained. Carvacrol is an aromatic monoterpene present in the essential oil extracted mostly from oregano, which exhibits antimicrobial properties.^21,35−38^ The MOF scaffold not only stabilizes and protects the active molecule but also promotes a unique delivery profile when supported in polymeric films, characterized by an unprecedented delay of an active agent and a prolonged release with an efficient antimicrobial effect (Figure 1). This exceptional performance makes carvacrol@MIL-100(Fe) a potential candidate for use in the food industry.

## Materials and Methods

### Materials

Iron(III) chloride hexahydrate (97%), 1,3,5-benzene tricarboxylic acid (95%), and carvacrol (98%) were purchased from Sigma-Aldrich, Alfa-Aesar, and TCI, respectively. Ethanol absolute was purchased from Honeywell. All chemicals were used as received without the need for further purification and Milli-Q water was obtained using a Millipore Milli-Q system.

### Synthesis of MIL-100(Fe) Nanoparticles

MIL-100(Fe) nanoparticles were synthesized following the procedure described by García Márquez and co-workers,^30^ by a microwave-assisted method. The as-synthesized material was treated in a KF 0.1 M solution for 2 h and washed with H_2_O to remove excess of reagents. The particles were collected by centrifugation (9900 rpm, 20 min) and stored.

### Preparation of the Carvacrol@MIL-100(Fe) Composite Material

A composite carvacrol@MIL-100(Fe) material was obtained following a direct impregnation method. Overall, 23 mL from a 10 mg/mL carvacrol emulsion prepared in a H_2_O/EtOH (4:1) mixture was directly added to MIL-100(Fe) nanoparticles (weighed humid and corresponding to 200 mg of dried MOF). After 5 days of stirring in a 360° rotating shaker, the carvacrol@MIL-100(Fe) material was retrieved by centrifugation (10 000 rpm, 20 min) and dried in air overnight.

### Determination of the Loading Capacity (LC) and Encapsulation Efficiency (EE)

The carvacrol content in the obtained composite was determined by thermal desorption gas chromatography, using an HP 7890B equipped with an HP5 column of 30 m, 320 μm diameter, and 0.25 μm thickness. The thermal gradient employed was 40 °C (3 min), 10 °C/min ramping until 200 °C, and 15 min isotherm. The injector was heated on a ballistic ramp (600 °C/min) from 40 to 200 °C and 4 min isotherm. Equations used for calculations of the loading capacity and encapsulation efficiency are further described in the Supporting Material.

### Characterization of the Carvacrol@MIL-100(Fe) Composite Material

Scanning electron microscopy images were acquired using a Hitachi S4800 microscope. For transmission electron microscopy, a JEM 1010 (JEOL) microscope was employed. X-ray powder diffraction experiments were acquired on an X-ray diffractometer (PANalytical Empyrean) with copper as radiation source (Cu Kα, 1.5418 Å). Infrared spectroscopy spectra were registered using an ALPHA II FTIR spectrometer (Bruker). Thermogravimetric analyses (TGA) were carried out on a TGA 550 (TA Instruments) in a high-resolution mode (Ramp: 20.0 °C/min to 680.00 °C; Res 4 Sensitivity 3). N_2_ sorption isotherms were obtained using a TRISTAR II apparatus (Micromeritics) at −196 °C. All samples were activated at 100 °C under vacuum for 3 h before measurement. Mössbauer spectra were collected using a conventional constant acceleration spectrometer and a ^57^Co (Rh) source. The velocity scale was calibrated with an α-Fe foil. Low-temperature measurements were performed with the samples immersed in He exchange gas in a bath cryostat. The spectra were fitted to distributions of quadrupole doublets according to the histogram method.^39^ Electron paramagnetic resonance (EPR) spectra were collected using a Bruker ELEXYS E580 spectrometer operating in the X-band (9.47GHz): sweep width, 4960.0 G; time constant, 2.56 ms; modulation frequency, 100 kHz; modulation width, 1 G; and microwave power, 19.82 mW.

### Preparation of Polymeric Films

Two different types of zein films were prepared. An 80% hydroalcoholic solution containing 15% weight of zein was first prepared. Two fractions were separated: to the first one, 0.125 g of the carvacrol@MIL-100(Fe) composite was added per g of zein, whereas the corresponding amount of carvacrol was directly added to the second fraction in a concentration of 0.05 g/g zein. The mixtures were spread on a flat surface of PTFE using a 200 μm coating rod and dried in an open-air oven at 75 °C for 10 min. The films obtained were stored in PP/met envelopes until further characterization. The residual content of carvacrol in both films was analyzed. For this, samples were cut from both films, introduced into a micro vial, and tested by thermal desorption and gas chromatography, obtaining a final concentration of carvacrol of 0.059 ± 0.008 g/g in the encapsulated film and 0.050 ± 0.001 g/g in the film with pure carvacrol.

### Carvacrol Release in the Films

Relative cumulative release profiles were measured by monitoring the carvacrol release from the polymeric films at 23 ± 1 °C and 95 ± 3% RH (simulating the exposure to fresh food). A piece sample of the film was placed on a desorption tube where a humid 15 mL/min stream of He hauled the released product to a gas chromatograph. The carvacrol release flow was determined using an HP5890 gas chromatograph with a 200 μL automatic injection valve and an HP5 column of 30 m, 320 μm diameter, and 0.25 μm thickness. Thermal conditions were as follows: 80 °C at the injection valve, 200 °C at the injection port, 220 °C at the flame ionization detector, and 100 °C at the column oven. Film samples were exposed to high humidity conditions with a He stream.

### Antimicrobial Properties of the Films

Bacterial strain *Escherichia coli* CECT 434 (ATCC 25922) and *Listeria innocua* CECT 910 (ATCC 33090), as subrogate strain for *Listeria monocytogenes*, were obtained from the Spanish Type Culture Collection (Valencia, Spain). These strains were chosen because of their relevance in the food industry as a Gram-negative and Gram-positive model, respectively. They were stored in a liquid medium tryptone soy broth (TSB) supplied from Scharlab (Barcelona, Spain) with 20% glycerol at −80 °C until needed. The stock cultures were maintained by periodic subculture on tryptone soy agar (TSA) from Scharlab (Barcelona, Spain) slants at 4 °C and transferred monthly. To test the antimicrobial effect of active films, the microatmosphere method, in which the volatile active compound released from the film to the headspace of the Petri dish interacts with the microorganisms, was carried out. In this method, 100 μL of a bacterial suspension containing approximately 10^7^ colony-forming units (CFU)/mL was spread over 15 mL of the TSA surface, and a disk of the antimicrobial films (80 mm in diameter) was adhered to the lid of the Petri dish, without direct contact with the microorganism, sealed with Parafilm and incubated at 37 °C for 24–48 h. After the incubation period, the diameter of the resulting inhibition zone in the bacterial growth was measured.^40^ Controls without films and control films with free carvacrol were also tested. Finally, the inoculated Petri dishes were employed to count the colony-forming units (CFU) and the log reduction value (LRV). For that, the agar medium was aseptically removed from the Petri dishes and homogenized in a sterile BagPage with 100 mL of peptone water for 2 min with a stomacher. Serial dilutions were made with peptone water and plated in Petri dishes with 15 mL of the selective agar medium: Brilliant Green agar for *E. coli* and supplemented Palcam agar for *Listeria innocua*, respectively, (Scharlab, Barcelona, Spain). Plates were incubated at 37 °C for 24–48h. Results were expressed as log CFU/mL. LRV was calculated by comparison between the control sample and samples with the film (LRV = (control log CFU/mL) – (film log CFU/mL)). The experiments were carried out in triplicate.

### Cell Culture

HEK293 human embryonic kidney cells were obtained from the American Type Culture Collection (ATCC), cultured in DMEM supplemented with 10% fetal bovine serum (FBS, Gibco), 1% penicillin/streptomycin (Sigma), and 0.1% amphotericin B (Gibco), and maintained in 20% O_2_ and 5% CO_2_ at 37 °C. Cells were routinely tested for mycoplasma using the universal mycoplasma detection kit (ATCC).

### Cell Viability Studies

HEK293 cells were plated on a 96-well plate (20 000 cells/well) and allowed to adhere to the wells. At 24 h post-seeding, the cells were incubated with varying concentrations of carvacrol and carvacrol@MIL-100(Fe) for 24 h. The cell viability was evaluated using the CellTiter 96 AQueous One Solution Cell Proliferation Assay (Promega). The absorbance was recorded at 450 and 570 nm 1 h later with a 96-well plate reader (Thermo Forma Fisher, Multiskan).

### Computational Details

Theoretical calculations were performed under the density functional theory (DFT) framework by means of the Gaussian-16.A03 suite of programs.^41^ Minimum-energy geometry structures were obtained upon atom relaxation at the B3LYP/6-31G(d,p) level of theory^42,43^ including dispersion corrections by means of Grimme’s D3 (Becke–Johnson damping function) protocol.^44,45^ To maintain the topology of the cluster models as in the corresponding MOF, the terminal hydrogen atoms were frozen during the optimization procedure. Non-covalent interaction (NCI) surfaces were obtained under the NCIPLOT-3.0 program^46^ and visualized through VMD software^47^ with standard thresholds of 0.3 and 0.04 au for the reduced density gradient and density, respectively. Time-dependent DFT calculations were performed on the lowest-lying singlet excited states at the B3LYP/6-31G(d,p) level. Charge analysis was performed through the natural bond order (NBO) approach by means of the NBO version 3 as implemented in Gaussian-16.A03. Spin density contours were plotted through Chemcraft software.^48^

## Results and Discussion

### Carvacrol Encapsulation in MIL-100(Fe) Nanoparticles

**Figure 2:**
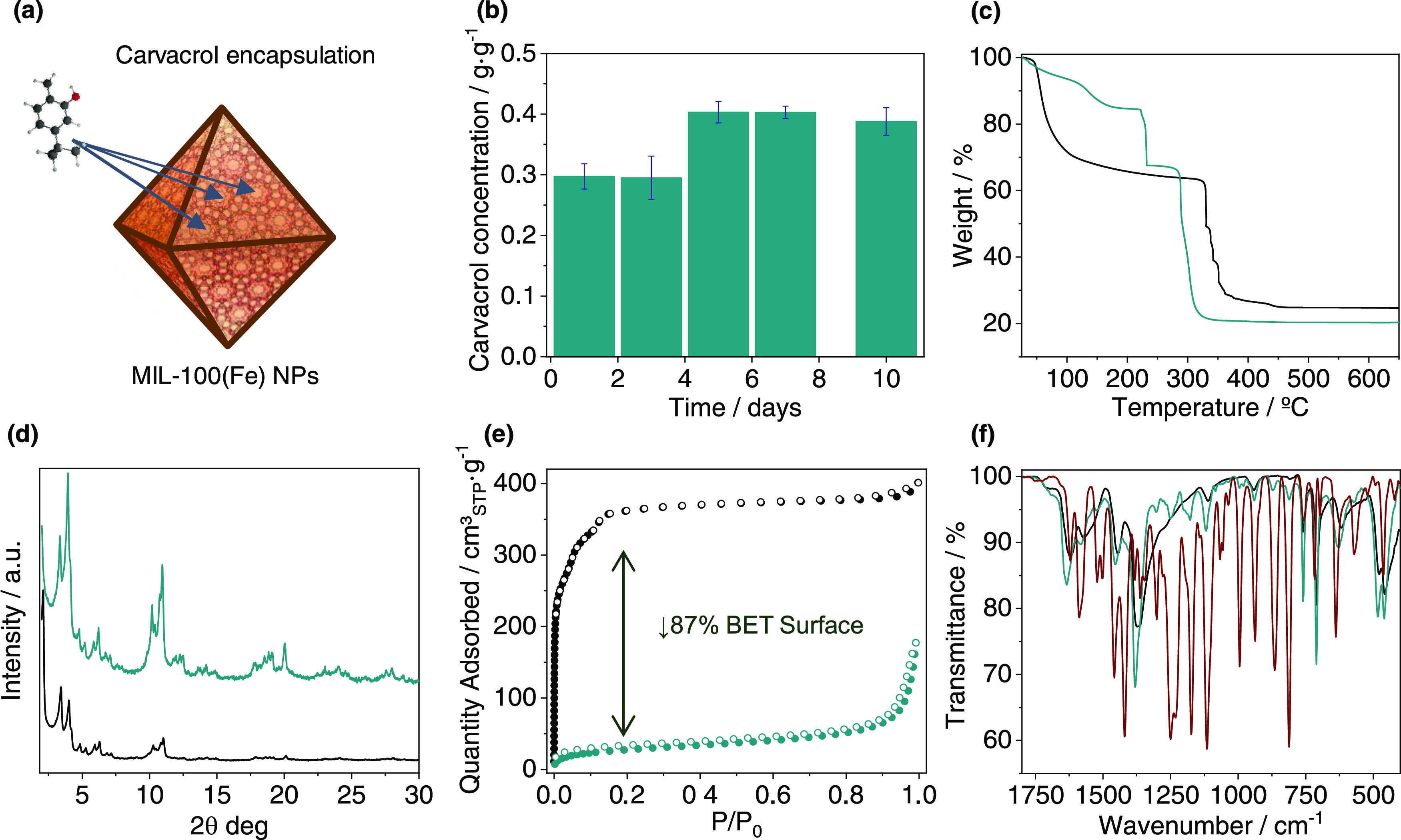
(a) Scheme of carvacrol encapsulation into MIL-100(Fe), (b) carvacrol content in the composite as a function of time of incubation, and (c–f) characterization of the carvacrol@MIL-100(Fe) composite (green) as compared with MIL-100(Fe) (black): (c) thermal decomposition profiles, (d) X-ray powder diffractograms, (e) N_2_ sorption studies at −196 °C (solid symbols for adsorption and open ones for desorption), and (f) selected infrared spectra region (1800–400 cm^–1^) of the composite as compared with MIL-100(Fe) and free carvacrol (red).

To encapsulate the natural food preserving molecule, carvacrol, we followed a simple green method consisting of the direct impregnation of previously obtained MIL-100(Fe) nanoparticles with a concentrated carvacrol solution (Figure 2a). Different experimental parameters including solvents and reaction times were investigated (Table S1). Best carvacrol encapsulation resulted from aqueous-alcoholic mixtures in which carvacrol is poorly soluble and forms a milky emulsion. Thus, carvacrol loading was achieved by soaking MIL-100(Fe) nanoparticles into a 10 mg/mL carvacrol ethanolic solution (20%) with a 5:1 carvacrol/MIL-100(Fe) molar ratio. First evidence of effective carvacrol loading was noticed by a color change in the powder from orange to dark brown, respectively, for bare MIL-100(Fe) and the collected carvacrol@MIL-100(Fe) composite (Figure S1). Encapsulation completion was determined upon monitoring of the carvacrol sorption equilibrium over time in the collected composite material by gas chromatography (Figure 2b). A maximum loading capacity of 42% was reached after 5 days of immersion, with no further loading occurring up to 10 days (% calculated as the loaded mass of carvacrol per total mass of the dry composite), resulting in an encapsulation efficiency of 58%. This exceptional payload is, to the best of our knowledge, the highest carvacrol uptake onto a MOF obtained in a liquid-phase encapsulation, surpassing the considerable 34% of carvacrol loading achieved in MIL-53(Al) by supercritical CO_2_ encapsulation.^49^ In addition, as compared to other classical carrier agents, the obtained carvacrol@MIL-100(Fe) composite is in a competitive position in terms of carvacrol loading, only being surpassed by carvacrol-loaded human serum albumin nanoparticles (Table S2).^50^

Carvacrol loading was further evaluated by thermogravimetric analysis (TGA), X-ray powder diffraction (XRPD), N_2_ sorption measurements, and Fourier transform infrared (FTIR) spectroscopy. Thermal analysis (Figures 2c and S2, Table S3) of the loaded carvacrol@MIL-100(Fe) composite revealed a carvacrol content of 26.7 wt % (with respect to dehydrated MIL-100(Fe) nanoparticles). After removal of volatiles, in the 120–180 °C interval and at 230 °C, two separated mass losses of 8.3 and 18.4% can be, respectively, distinguished, which may be attributed to the release of carvacrol molecules differently interacting with the framework (i.e., physisorption vs chemisorption).

MOF integrity was evaluated after carvacrol infiltration by means of XRPD analysis performed before and after the encapsulation process (Figure 2d), confirming that the MIL-100(Fe) crystal structure is maintained after carvacrol loading. This was also confirmed by SEM and TEM images (Figures S3 and S4), which corroborate that the morphology of the particles remains unaffected by the encapsulation process. Then, to identify the porous nature of the composite material and therefore determine the external or internal association of the carvacrol molecules, N_2_ sorption studies were conducted before and after carvacrol impregnation (Figure 2e). A significant decrease (ca. 87%) in the porous surface was evidenced after carvacrol encapsulation (*S*_BET_ = 1491 vs 196 m^2^/g, respectively, for MIL-100(Fe) and carvacrol@MIL-100(Fe)), which is consistent with an effective carvacrol loading.

**Figure 3:**
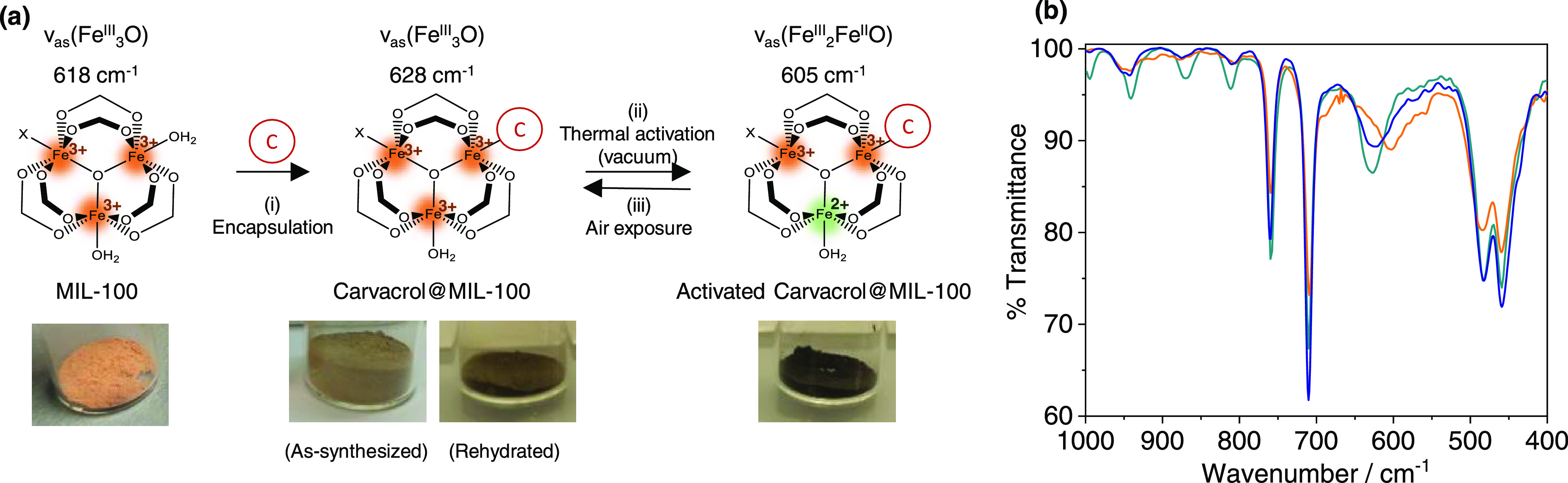
(a) Scheme of (i) encapsulation, (ii) thermal activation under vacuum, and (iii) reversible air exposure processes, with the corresponding color change in each step. The position of the IR asymmetric stretching bands of the iron trimeric unit is highlighted. (b) Selected infrared spectra comparison of the composite before activation (green), after thermal treatment under vacuum (190 °C for 2 h) (orange), and after air exposure for 1 min (blue).

Analysis of the FTIR spectra of the impregnated carvacrol@MIL-100(Fe) material (Figures 2f and S5) revealed the appearance of the most remarkable bands of carvacrol (ν_O–H_ 3361 cm^–1^ and ν_C–H_ 2958 and 2869 cm^–1^) in addition to the typical bands corresponding to Fe^III^ trimesate MOF (ν_C=O_ 1620, 1570, 1445, and 1370 cm^–1^). Moreover, it can be noticed that the ν_as_(Fe_3_O) vibration at 618 cm^–1^, characteristic of the Fe^III^ oxo-trimeric core in MIL-100(Fe),^51^ appears shifted to 628 cm^–1^ in the composite material. This suggests an effective interaction between the carvacrol molecules and the accessible Fe^III^ sites, as previously described for the binding of different molecules.^52,53^ In an attempt to further investigate the host–guest interaction, the biocomposite and the control empty material were exposed to thermal treatment under vacuum for 2 h. Thermal activation (250 °C) has been previously shown to induce the generation of coordinatively unsaturated iron sites (CUS) with mixed-valence Fe^II^/Fe^III^ trimers in MIL-100(Fe), manifested by a shift of the ν_as_(Fe_3_O) band located at 618 to 597 cm^–1^ after thermal treatment in IR spectroscopy studies.^33,51^ In a similar study, we limited this high-temperature activation to 190 °C to remain below the carvacrol boiling point. Exposing MIL-100(Fe) control to this thermal activation leads to a reduced shifting in the ν_as_(Fe_3_O) from 618 to 611 cm^–1^, recovering the original value upon 1 min of air exposure (Figure S6). This band evolution reflects a reversible partial loss of coordinated water molecules as compared to the reported material, as may be expected for lower activation temperature and lack of sample isolation from ambient conditions.^33,51^ In the case of carvacrol@MIL-100(Fe) treated at 190 °C under vacuum, a shift in the ν_as_(Fe_3_O) band from 628 to 605 cm^–1^ is observed, and the original value is recovered after 1 min of air exposure (Figure 3). The large shifting of the ν_as_(Fe_3_O) band observed in the case of the activated composite is in good agreement with the displacement reported in the mixed-valence Fe^II^/Fe^III^ MIL-100(Fe) spectrum (23 vs 21 cm^–1^, respectively). This phenomenon is accompanied by a drastic color change from brown to an intense black carbon-like color, this color change being reversible to some extent upon exposure to air. It is worth mentioning that crystallinity was preserved during this process, as denoted by the maintenance of the XRPD patterns in the material (Figure S7).

### Mössbauer and EPR Spectroscopies

**Figure 4:**
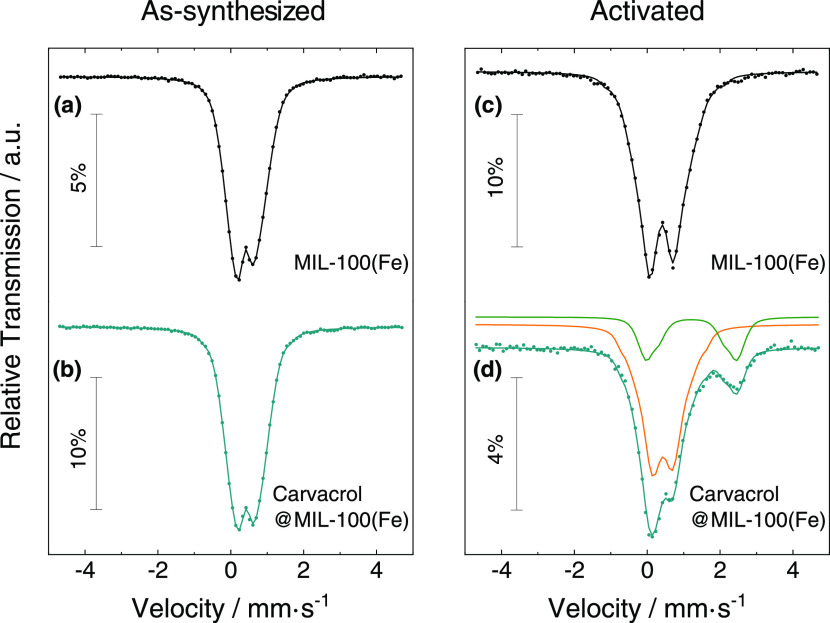
Mössbauer spectra recorded at 80 K of (a) MIL-100(Fe) and (b) carvacrol@MIL-100(Fe) measured as-synthesized and (c) MIL-100(Fe) and (d) carvacrol@MIL-100(Fe) measured after activation (right). The solid lines over the experimental points are the fitted distribution of quadrupole doublets. The sum of two distributions of quadrupole doublets due to Fe^III^ and Fe^II^ is shown slightly shifted for clarity in orange and green, respectively.

To evaluate the effect of carvacrol loading in the MIL-100(Fe) electronic structure, Mössbauer spectroscopy experiments were carried out. All spectra were collected at 80 K. Control experiments in the as-synthesized MIL-100(Fe) and carvacrol@MIL-100(Fe)-loaded materials revealed spectra with two broad peaks fitted to distributions of quadrupole doublets (Figure 4). Table S4 summarizes the calculated isomer shift (IS) values as well as the temperature-independent quadrupole splitting (QS), which are consistent with high-spin Fe^III^ (*S* = 5/2) in both samples.^30,54−56^ In the case of the carvacrol@MIL-100(Fe) composite, a lower QS of Fe^III^ can be detected, which suggests a different Fe^III^ environment in the composite. This difference may arise from the replacement of one of the water molecules coordinated to the Fe^III^ centers by one molecule of carvacrol, in agreement with the shifting of the ν_as_(Fe_3_O) band observed in IR measurements. Studies on the activated compounds were then performed after exposing MIL-100(Fe) and carvacrol@MIL-100(Fe) samples to thermal treatment (190 °C) under vacuum for 6 h, and quenching the samples in liquid nitrogen for analysis. As expected, the activated MIL-100(Fe) spectrum obtained at 80 K (Figure 4c) showed significantly higher average QS as compared to the pristine sample. This is consistent with a more distorted Fe^III^ environment becoming 5-coordinated due to the loss of coordinated water molecules.^33^ Once the sample was brought back to room temperature in air, the spectrum of the untreated sample was recovered, confirming the reversibility of the desolvation process. A similar analysis performed on the activated carvacrol@MIL-100(Fe) spectrum (Figure 4d) indicates that approximately 24% of the Fe is reduced to high-spin Fe^II^, with the estimated IS and QS suggesting that both Fe^II^ and Fe^III^ are 6-coordinated.^54^ Then, the initial carvacrol@MIL-100(Fe) spectrum is recovered after exposing the activated sample in air and at room temperature for a few hours. These observations are in agreement with IR experiments and denote the effective reversible formation of the mixed-valence trimer in the composite material with retention of the carvacrol molecules coordinated to the Fe^III^ centers.

To further investigate the coordination between carvacrol and MIL-100(Fe), EPR experiments were conducted. Spectra recorded at room temperature revealed similar signals for the as-synthesized materials before and after encapsulation (Figure S8), whereas the appearance of a characteristic radical signal (*g* = 2.005) was observed upon activation of the composite (190 °C under vacuum for 2 h), suggesting a radical-mediated mechanism for the carvacrol coordination to the Fe^III^ centers. Interestingly, this radical remains stable with time, as long as the material is isolated from air.

### Theoretical Calculations

**Figure 5:**
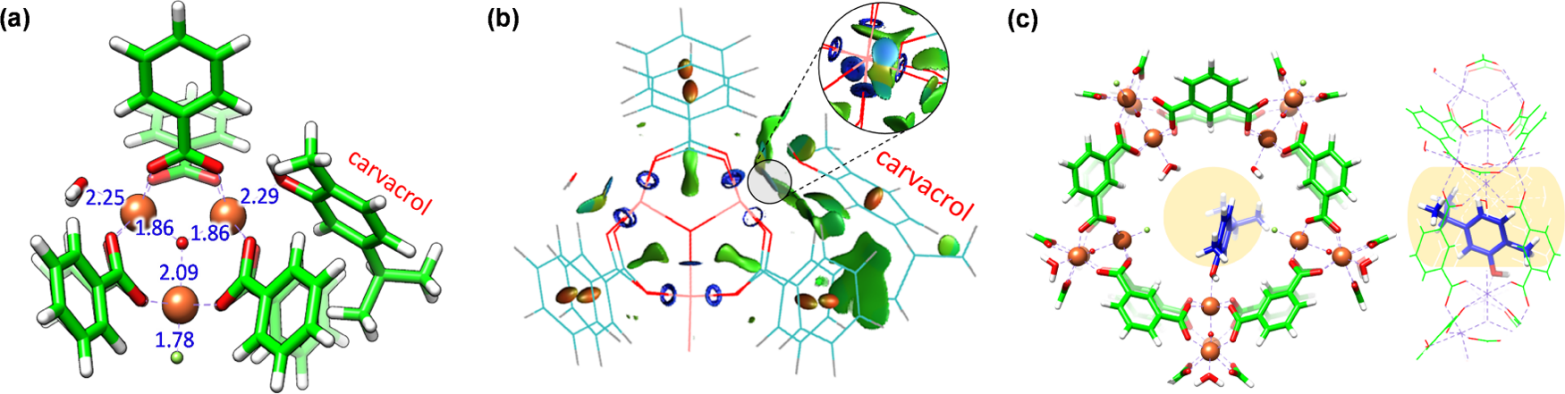
(a) Minimum-energy structure for the representative cluster of MIL-100(Fe) upon carvacrol inclusion. Relevant coordination distances are indicated in Å. Color coding: C in green, O in red, H in white, and Fe in orange. (b) Noncovalent index (NCI) surface showing the large amount of dispersion interactions that stabilize the chemisorption of carvacrol into the MIL-100(Fe) cluster. (c) Front (left) and side (right) views of the minimum-energy structure calculated for the MIL-100(Fe) pentagonal window upon carvacrol complexation. Carvacrol carbon atoms are colored in blue for a better view. The window gate is calculated with a diameter of 5.5 Å and colored in light yellow.

Density functional theory calculations were performed to shed light on the coordination process between carvacrol and the Fe^III^ oxo-trimeric core, and the formation of the mixed-valence Fe^II^/Fe^III^ MIL-100(Fe) upon thermal activation. An oxo-centered trimetallic cluster model with six benzoate ligands was extracted from the crystal structure of MIL-100(Fe), bearing two water molecules and a fluoride anion in the coordination environment of Fe^III^ atoms for charge neutrality, and was optimized at the B3LYP/6-31G(d,p) level of theory in a high-spin configuration (Figure S9). A preliminary evaluation of the thermodynamics of Fe^III^ coordination bonds indicates that insertion of carvacrol in MIL-100(Fe) is expected to occur by replacing a coordinative water molecule (Figure S10); this process is energetically favored (Δ*E* = −16.15 kcal/mol) (Figure S11). The minimum-energy structure of the oxo-centered trimetallic cluster indicates that the hydroxy group of carvacrol effectively coordinates with an Fe^III^ atom, displaying a small interatomic distance of 2.29 Å (Figure 5a), similar to that calculated for a coordinating water molecule (2.25 Å). Noncovalent index (NCI) surfaces evidence a large number of weak but stabilizing dispersion interactions between the carvacrol moiety and the benzoate units of the cluster (green surfaces in Figure 5b), whereas the strong coordination bond between the hydroxy group of carvacrol and one Fe^III^ is revealed as a localized bluish NCI surface (Figure 5b inset and Figure S12).

A bigger cluster consisting of the pentagonal gate of MIL-100(Fe) was modeled to support the structural and stability properties predicted for the coordination of carvacrol. After geometry optimization, a carvacrol molecule was placed inside the pentagonal window to replace a coordinating water molecule (Figure 5c). Theoretical calculations at the B3LYP/6-31G(d,p) level indicate that the coordination of carvacrol is significantly more stable than with water (Δ*E* = −11.23 kcal/mol for the reaction of replacing a coordinating water by carvacrol), in good accord with that predicted for the smaller cluster model and in line with the experimental evidence. NCI surfaces confirm the presence of a large number of noncovalent interactions between the carvacrol molecule and the framework, along with a strong, localized interaction corresponding to the coordination bond with Fe^III^ (see Figure S13). Note that the pentagonal pore gate is predicted with a diameter of 5.5 Å, which nicely fits the carvacrol size of 5.0 Å calculated without considering the hydroxy group (see the light yellow region in Figure 5c). The hexagonal pore gate, which is of about 8.6 Å, is therefore expected to bear and diffuse the carvacrol along the framework easily.

**Figure 6:**
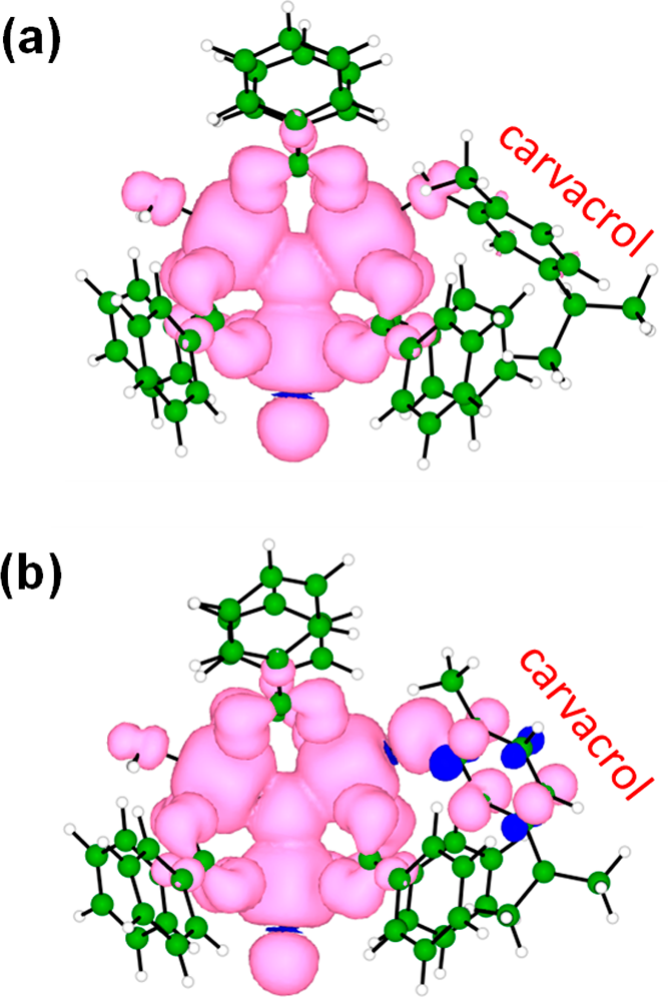
Spin density contours (isovalue = 0.001 au) calculated for the representative trimetallic MIL-100(Fe) cluster with coordination of the (a) carvacrol molecule or (b) deprotonated carvacrol (radical) system.

Spin density contours indicate that coordinated carvacrol, in its neutral protonated form, does not show a radical character (oxidized state) that could explain the reduction of one Fe^III^ to engender a mixed-valence Fe^II^/Fe^III^ system (Figure 6a). Accumulated charges confirm the closed-shell nature of the carvacrol moiety, which is barely charged (+0.08e; Figure S14), similar to that calculated for the coordinated water molecule (+0.09e). On the other hand, time-dependent DFT calculations predict low-lying singlet electronic transitions in the range of 2.2–2.6 eV from the carvacrol system to the Fe-d orbitals (Figure S15), supporting its electron-donor character compared to the trimetallic cluster of MIL-100(Fe). Spontaneous one-electron carvacrol oxidation and MIL-100(Fe) reduction were hypothesized to originate upon deprotonation of the relatively acid hydroxy group of carvacrol when coordinated. Theoretical calculations show that proton transfer to other entities is energetically feasible (for example, proton transfer to a coordinating fluoride and water exchange is calculated <10 kcal/mol; Figure S16). Upon carvacrol deprotonation, the excess of charge in the carvacrol moiety is partially withdrawn by the oxo-centered cluster, especially in one of the iron atoms, thus supporting the formation of a mixed-valence Fe^II^/Fe^III^ trimetallic system (Figure S14). This leads to the formation of a carvacrol radical, which is confirmed by the spin density contours (Figure 6b). The radical carvacrol species coordinated to MIL-100(Fe) is predicted to exhibit several low-lying singlet excited states of charge-transfer nature, with a moderate intensity, and extending over the full visible region of the absorption spectrum (Figure S17). These results are in agreement with the black carbon-like color displayed by carvacrol@MIL-100(Fe) upon thermal activation.

### Release Kinetics of Encapsulated Carvacrol from Films

**Figure 7:**
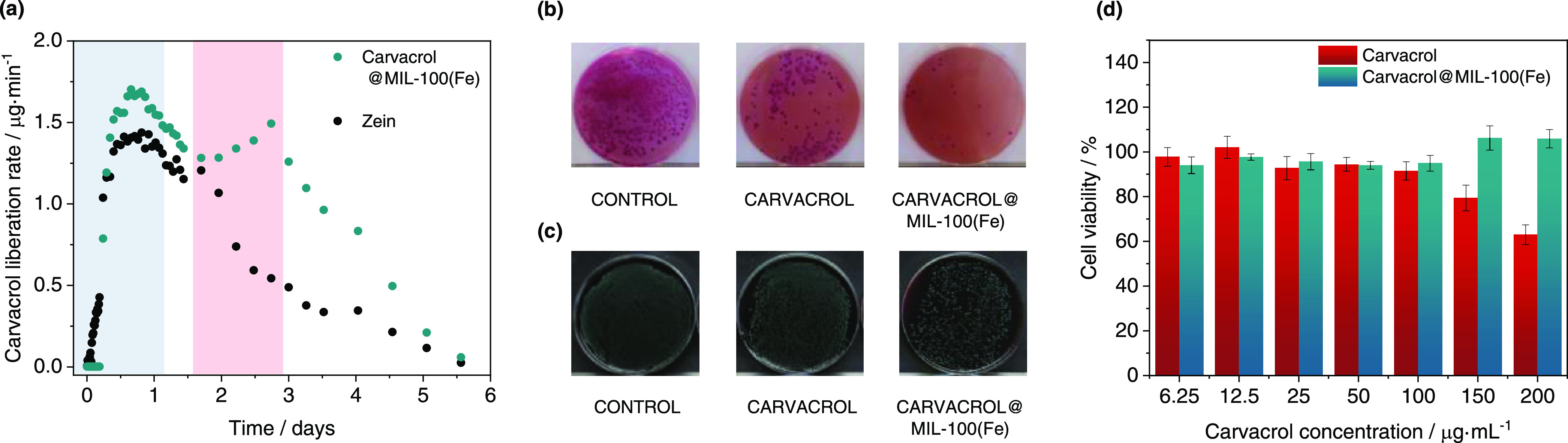
(a) Carvacrol liberation rate in 1 g of the zein film containing the carvacrol@MIL-100(Fe) composite. (b, c) Images of the microatmosphere test carried out to determine the activity of films against (b) *E. coli* and (c) *L. innocua*: images of the Petri dishes showing the grass density generated by bacteria after the incubation for control (left), the carvacrol film (middle), and the carvacrol@MIL-100(Fe) containing film (right) with Brilliant Green agar after the 5th decimal dilution (b) and with Palcam agar after the 3rd decimal dilution (c). (d) Cell viability of HEK293 cells in contact with free carvacrol (red) and the carvacrol@MIL-100(Fe) biocomposite (green) at carvacrol concentrations between 6.25 and 200 μg/mL for 24 h. The percent viable cells were calculated in comparison to untreated cells taken as 100%.

Carvacrol liberation was studied after the incorporation of the composite in a zein polymeric matrix simulating fresh food environmental conditions (see the Materials and Methods section). Figure 7a shows the results of carvacrol liberation from the composite-containing film as compared to a control film containing free carvacrol in the same concentration. The control free carvacrol release profile is characterized by a direct continuous delivery with a maximum at 20 h followed by an exponential decay, which corresponds with a delivery governed by diffusion until the depletion of the active agent. A unique profile is observed in the composite-containing film, which is described with a significant carvacrol retention of ca. 5 h occurring before the liberation starts. After 5 h, two clearly different delivery phases are observed that are ascribed to the occurrence of sequential fast and slow desorption processes reflected in a first maximum at 20 h, followed by an additional second maximum at 66 h. This behavior can be explained by attributing each maximum to different host–guest interactions between the carvacrol molecules and the MIL-100(Fe) framework. The first release may correspond to weakly interacting carvacrol molecules, whereas up to 2 days of high relative humidity conditions are required to trigger the liberation of the chemisorbed molecules. This MOF-mediated two-step release represents a clear improvement as compared to the same amount of free molecule in the polymeric film, evidencing the unique MOF capability to deliver carvacrol at prolonged times.

Microbiology studies were conducted to determine the efficiency of the obtained composite against *E. coli* and *L. innocua* (as a subrogate for *L. monocytogenes*). These different species have been selected considering the antibacterial mechanism of phenolic carvacrol molecules, which are known to interact with the lipid bilayer that constitutes the cytoplasmic bacterium membrane, with a subsequent increase of its permeability.^57^ To analyze the antimicrobial effect of the volatile agent in the vapor phase, we used the microatmosphere method reported by some of us.^40^ This method consists of producing a bacterial background lawn on the surface of the agar and evaluating the inhibition effect of the vapor delivered from the film on the density of this lawn. Three dishes were seeded with each bacterium, one containing a film of the carvacrol@MIL-100(Fe) composite, the second containing a zein film with the same concentration of free carvacrol, and the third one as a control. It is important to note that a high concentration of bacteria (10^7^ CFU/mL, where CFU stands for colony-forming units) is needed to obtain an adequate bacterial lawn. Then, the agar-inoculated medium was aseptically removed and homogenized to recover bacteria and bacterial counts. The optimum time and incubation temperature allowed the microbial growth to reach 9 log in control samples.

Figures 7b,c reveal a clear reduction of the grass density occurring on the samples with carvacrol, corroborating the antimicrobial effect of the active films, an effect that is particularly more evident with the films containing the carvacrol@MIL-100(Fe) composite. Table 1 summarizes the quantitative effects of the active films against *E. coli* and *L. innocua* bacteria. In the case of *E. coli*, a clear CFU reduction of 1.02 and 1.76 was, respectively, obtained for free and MOF-encapsulated carvacrol films, which translates into an 82% increase in the carvacrol antimicrobial effect upon encapsulation into MIL-100(Fe). This effect was also evidenced in *L. innocua* bacteria, where free carvacrol was able to reach a CFU reduction of 0.40, while the CFU reduction of carvacrol@MIL-100(Fe) was 1.57, achieving a 93% increase in the antimicrobial effect as compared with the free carvacrol in the film. This effectiveness against *L. innocua* is particularly relevant, provided the demonstrated reduced activity of carvacrol against Gram-positive bacteria.^49^

**Table 1:** Antimicrobial Activity of Films (80 mm Diameter Surface) against *E. coli* and *L. innocua*^a^

sample	*E. coli*	LRV	*L. innocua*	LRV
control	9.04 ± 0.12		9.21 ± 0.15	
free carvacrol film	8.02 ± 0.21	1.02	8.81 ± 0.14	0.40
carvacrol@MOF composite film	7.28 ± 0.11	1.76	7.64 ± 0.87	1.57

a Data are shown as log (colony-forming units/mL) and the log reduction value (LRV) observed after incubation in Petri dishes without the film (control), the free carvacrol film, and the film containing the carvacrol@MOF composite.

This greater antibacterial effect observed in the film with encapsulated carvacrol is potentially bounded to two significant effects: (i) a larger content of carvacrol and (ii) the different releases from the film in the composite material. Thus, despite initially incorporating an equivalent carvacrol amount in the control and composite samples, the final content of carvacrol was ca. 20% greater in the carvacrol@MIL-100(Fe) film. This carvacrol difference may be produced during film drying, which highlights the capacity of the nanoMOF to retain the carvacrol molecules. It should be also noticed that the Petri dish is not a hermetic container since bacteria need to breathe. Under these conditions, different carvacrol losses through the openings between the dish and the lid and by scalping on the dish matrix (made of polystyrene) may also contribute to some extent to differences in the final content of carvacrol. Considering the different release, a fast release is observed in the samples with free carvacrol, with an initial high concentration of carvacrol that rapidly decreases. On the contrary, encapsulated carvacrol@MIL-100(Fe) retains carvacrol up to 5h and then releases it at a slower pace, providing a sustained antibacterial effect along the assay period. This effect constitutes an advantage over other carriers since the abrupt initial release of active molecules in the first hours may not be required under specific uses such in fresh product preservation, provided that bacterial growth has not started yet. Both properties, a larger carvacrol content and a delayed sustained release are unique characteristics of our carvacrol@MIL-100(Fe) composite that result in a more efficient antimicrobial effect of carvacrol against *E. coli* and *L. innocua*.

To study the cellular toxicity after prolonged exposure to the carvacrol@MIL-100(Fe) composite, HEK293 human embryonic kidney cells were used in cell viability assays at concentrations up to 200 μg/mL (Figure 7d). As expected, the use of free carvacrol reduces the cell viability by 40% at concentrations of 200 μg/mL, which is consistent with reported data showcasing cellular death if the carvacrol concentration surpasses 100 μg/mL.^58^ On the contrary, carvacrol@MIL-100(Fe) exhibits biocompatibility over a significantly wider range, observing that 100% of the cells were viable after exposure of carvacrol@MIL-100(Fe) containing a 200 μg/mL equivalent carvacrol amount for 24 h. Thus, it can be concluded that carvacrol encapsulation into MIL-100(Fe) minimizes the toxicity associated to large concentrations of free carvacrol, likely due to its ability to control the release of the active molecule into the incubation medium.

## Summary and Conclusions

A carvacrol@MIL-100(Fe) biocompatible composite containing considerable payloads of the active agent was prepared following a direct impregnation method compatible with food-related uses. In addition to providing chemical stability to the active molecule, the MIL-100(Fe) scaffold endorses an unprecedented retained and remarkable sustained delivery when processed in polymeric films because of its unique redox responsiveness that promotes effective interactions with the active agent. Mössbauer spectroscopy supported by theoretical calculations revealed a successful reversible interaction of the carvacrol molecules with the redox-active MIL-100(Fe) scaffold, thus enabling a prolonged delivery. Exposing the obtained composite under simulated fresh food conditions produced a released carvacrol dose enough to fight bacterial pathogens, with an improved activity against *E. coli* and *L. innocua*in comparison with an equivalent “free” carvacrol dosage.

The combination of a direct preparation, facile processing, and the MOF-mediated delivery performance that enables prolonged carvacrol bactericidal activity makes the obtained carvacrol@MIL-100(Fe) composite a promising candidate for food packaging applications.

## Supporting Information Available

Additional experimental details and methods and complementary physicochemical characterization of the carvacrol@MIL-100(Fe) composite, including photographs of the analyzed samples (PDF)

## Abbreviations

BAC: bioactive compounds
MOF: metal–organic framework
CUS: coordinatively unsaturated sites
EO: essential oil
EE: encapsulation efficiency
LC: loading capacity
GC: gas chromatography
TEM: transmission electron microscopy
XRPD: X-ray powder diffraction
FTIR: Fourier transform infrared
TGA: thermogravimetric analysis
EPR: electron paramagnetic resonance
RH: relative humidity
CFU: colony-forming units
LRV: log reduction value
TSB: tryptone soy broth
TSA: tryptone soy agar
