# Supplementary material for: Exploiting the Redox Activity of MIL-100(Fe) Carrier Enables Prolonged Carvacrol Antimicrobial Activity
Source: ACS Appl Mater Interfaces. 2022 Feb 18;14(8):10758–68. doi: 10.1021/acsami.1c21555 (PMC8895383; doi:10.1021/acsami.1c21555)
Supplement: Supplementary file 1 — am1c21555_si_001.pdf [file am1c21555_si_001.pdf]

Supporting Information

Exploiting redox activity of MIL-100(Fe) carrier enables carvacrol prolonged antimicrobial activity

Katia Caamaño,[a] Raquel Heras-Mozos,[b] Joaquín Calbo,[a] Jesús Cases Díaz,[a] João C. Waerenborgh,[c] Bruno J. C. Vieira,[c] Pilar Hernández-Muñoz,[b] Rafael Gavara[b] and Mónica Giménez-Marqués[*a].*

a. Instituto de Ciencia Molecular (ICMol), Universidad de Valencia, c/ Catedrático José Beltrán 2, 46980 Paterna, Spain.

b. Instituto de Agroquímica y Tecnología de Alimentos, IATA-CSIC, Av. Agustín Escardino 7, 46980 Paterna, Spain.

c. C2TN, DECN, Instituto Superior Técnico, Universidade de Lisboa, EN10, P-2695-066 Bobadela LRS, Portugal.

Corresponding Author

e-mail: monica.gimenez-marques@uv.es

Content

Carvacrol encapsulation **3**
Encapsulation efficiency 3
Physico-chemical characterization of Carvacrol@MIL-100(Fe) composite **7**
Thermogravimetric analysis 7
Field emission scanning electron microscopy 9
Transmission electron microscopy 9
Infrared spectroscopy 11
X-Ray Powder Diffraction 13
Mössbauer spectroscopy 14
Electron paramagnetic resonance spectroscopy 15
Theoretical calculations **16**

Carvacrol encapsulation

Encapsulation efficiency

Different synthetic parameters were investigated to optimize the encapsulation process. EtOH was initially selected due to the optimal carvacrol solubility in this medium. Different Carvacrol/MIL-100(Fe) molar ratios, mainly 1/1, 3/1 and 5/1, were explored, and the incubation was monitored up to 90 days. Chemical characterization of the composites (IR, TGA and N_2 sorption) evidenced that the encapsulation was not successful in this medium.

Attempts with different solvents were then explored obtaining best results in H_2O:EtOH (4:1) mixture of solvents. It is important to remark that under these conditions a white emulsion was obtained due to the limited solubility of carvacrol in water. Three parallel encapsulations were carried out in this medium, and stopped at 10, 20 and 30 days in order to monitor the loading. It was observed that encapsulations at 20 and 30 days were not significantly different from the 10-day encapsulation. Loading kinetics were then analyzed at 1, 3, 5, 7, and 10 days by thermic desorption Gas Chromatography. It was concluded that maximum carvacrol uptake occurred after 5 days of encapsulation, with no further loading occurring up to 10 days. Different carvacrol/MIL-100(Fe) molar ratios ranging from 1/1 to 10/1 were also studied up to 5-days encapsulations, in order to confirm that the selected parameters were optimal.

TABLE S1: Evaluated parameters in the optimization of the encapsulation protocol.

Solvent	Carvacrol/MIL-100(Fe) molar ratio	Encapsulation time (days)
**EtOH**	1/1	Aliquots up to 90 days of encapsulation
	3/1	
	5/1	
**H_2O/EtOH (4/1)**	5/1	10, 20, 30
		1, 3, 5, 7, 10
	1/1	5
	2/1	
	3/1	
	5/1	
	10/1	

After the screening of the solvent effect and carvacrol/MIL-100(Fe) molar ratio, best results were obtained with H_2O/EtOH (4/1) as a solvent and a molar ratio of 5/1.

Encapsulation temperature was also evaluated by performing two 5-day encapsulations with a molar ratio of 5/1 at room temperature and 37 °C in parallel, with no differences observed in encapsulation efficiency.

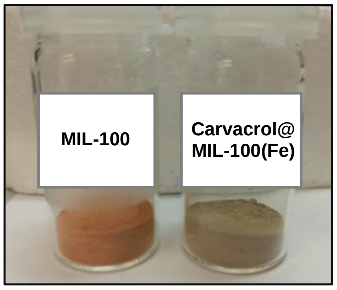

FIGURE S1: Colour difference between the pristine MIL-100(Fe) and the final biocomposite after the encapsulation process.

Loading capacity and **encapsulation efficiency** are defined according to the equations:

$$\text{L.C.\%} = \frac{mass\ of\ carvacrol}{mass\ of\ composite} \times 100$$

$$\text{E.E.\%} = \frac{mass\ of\ loaded\ carvacrol}{mass\ of\ inital\ carvacrol} \times 100$$

TABLE S2: Carvacrol encapsulation efficiency of the composite in comparison with classical carriers.

Encapsulation agent	Encapsulation efficiency (%)	Carvacrol loading (%)	Reference
**This work**	58	42	-
**Chitosan NPs**	14–31	3–21	*Colloids Surfaces B Biointerfaces*, **2011**, *84*, 163–171
**Poly(DL-lactide-co-glycolide) (PLGA) NPs**	26	21	*Int. J. Mol. Sci.*, **2011**, *12*, 5039–5051
**Hydroxypropyl-beta-cyclodextrin microcapsule**	83, 91	-	*Food Sci. Technol.*, **2015**, *60*, 583–592
**Human serum albumin NPs**	48	45	*IET Nanobiotechnology*, **2015**, *9*, 294–299
**Bovine serum albumin NPs**	68	27	*Iran. J. Pharm. Res.*, **2019**, *0*, 312–320
**Polyhydroxybutyrate NPs**	21	13	*J. Food Sci.*, **2014**, *79*, N697–N705
**Attapulgite (zeolite)**	76*	23	*J. Porous Mater.*, **2020**, *27*, 843–853

* Extracted from experimental data in published work.

Physico-chemical characterization of Carvacrol@MIL-100(Fe) composite

Thermogravimetric analysis

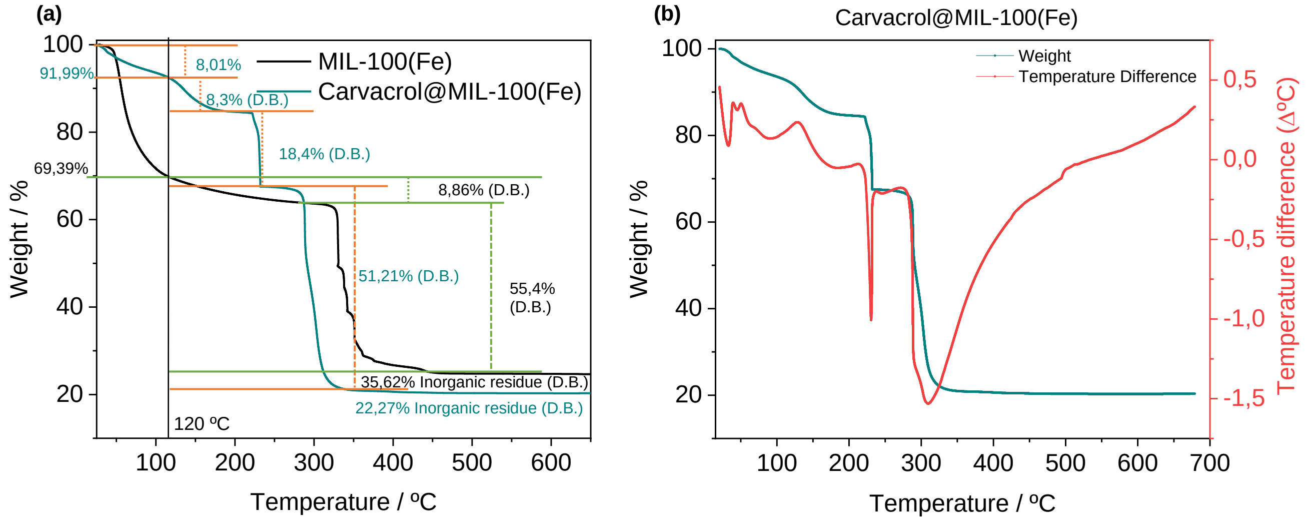

FIGURE S2: (a) TGA profiles of MIL-100(Fe) and the encapsulated material, with experimental weight percentages corresponding to mass losses. (b) Overlapping of the TGA profile of the composite material with the temperature difference in the mass drops.

TABLE S3: Mass loss attribution of the MOF and the composite material.

MIL-100(Fe)			Carvacrol@MIL-100(Fe)		
Range	Attributed to	Mass loss	Range	Attributed to	Mass loss
0-120ºC	Solvent	30.61%	0-120ºC	Solvent, superficial carvacrol	8.01 %
120-320ºC	Coordinated H2O	8.86%*	120-220ºC	Physisorbed carvacrol	8.3%*
320-500ºC	Ligand	55.40%*	220-250ºC	Chemisorbed carvacrol	18.4%*
500-700ºC	Inorganic residue	35.62%*	250-500ºC	Ligand	51.21%*
			500-700ºC	Inorganic residue	22,27%*

*Calculated as dry based.

Field emission scanning electron microscopy

Particle morphology of the materials was evaluated by means of field emission scanning electron microscopy. Samples were prepared by placing the powder in a carbon tape support and were subjected to metallization before collecting the images.

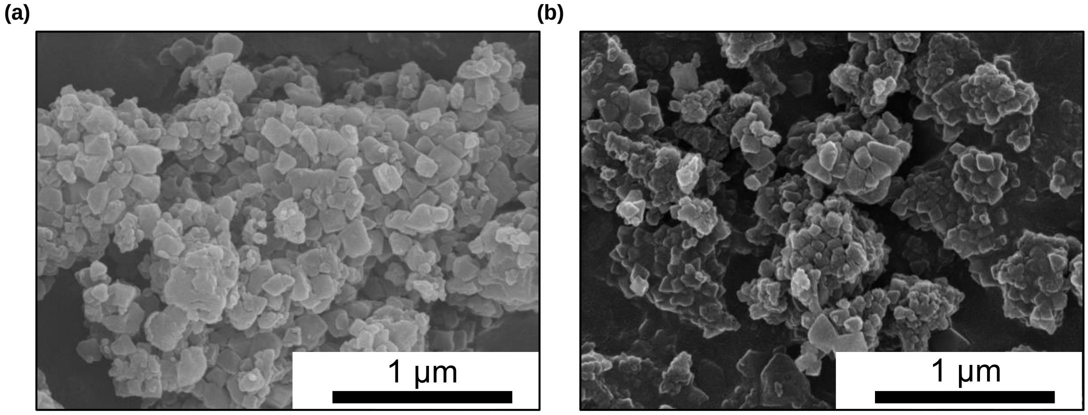

FIGURE S3: SEM images of (a)MIL-100(Fe) NPs and (b) Carvacrol@MIL-100(Fe) samples. Scale bar is 1 μm.

Transmission electron microscopy

Particle morphology of the materials was also evaluated by electron transmission microscopy. 15 μL of a 1 mg·mL^{-1} suspension in H_2O of each compound was dropped in a TEM grid and the grids were left to dry at air before collecting the images.

(a) (b)

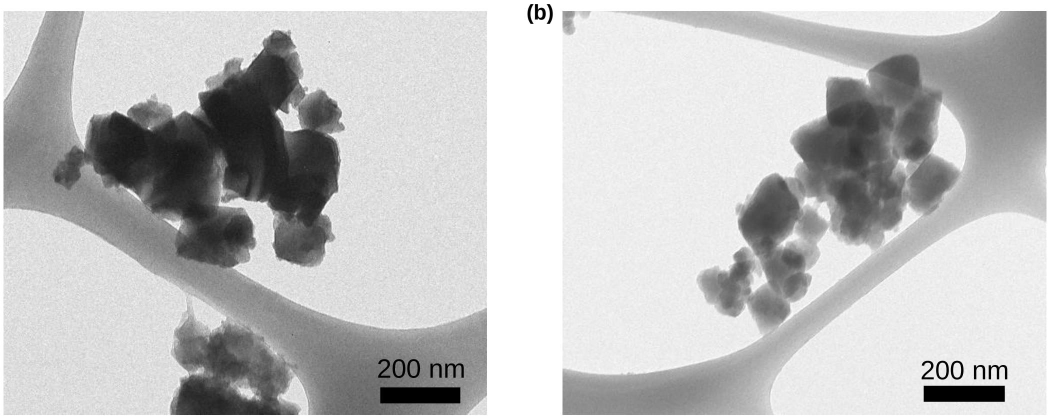

FIGURE S4: TEM images of (a)MIL-100(Fe) NPs and (b) Carvacrol@MIL-100(Fe) samples. Scale bar is 200 nm.

Infrared spectroscopy

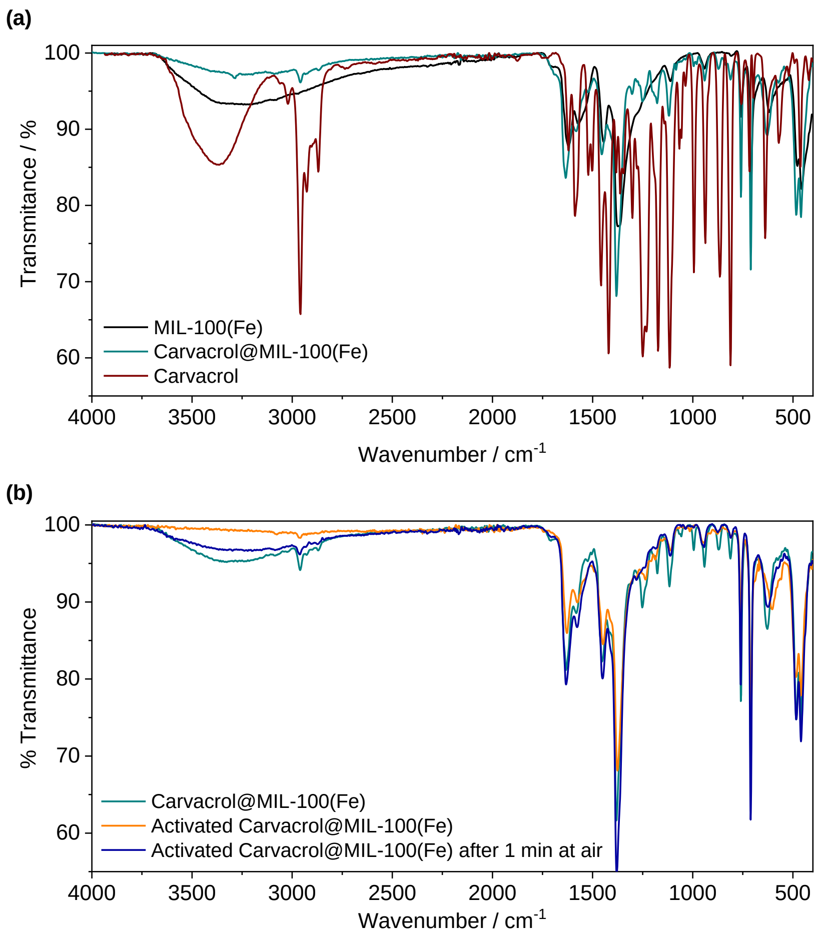

FIGURE S5: (a) IR spectra comparison of the composite (green) as compared with MIL-100(Fe) (black) and free carvacrol (red). (b) IR spectra of the composite material before (green) and after (orange) activation, compared with the activated composite upon 1 minute of air exposure (blue).

Monitoring of IR spectra upon thermal-vacuum cycles: MIL-100(Fe) and carvacrol@MIL-100(Fe) samples were introduced in a previously heated binder at 190 °C

and vacuum was applied. After 2 hours, the samples were immediately measured, and their spectra were collected. The samples were left at air on the sample holder for 1 minute and their spectra were recorded again.

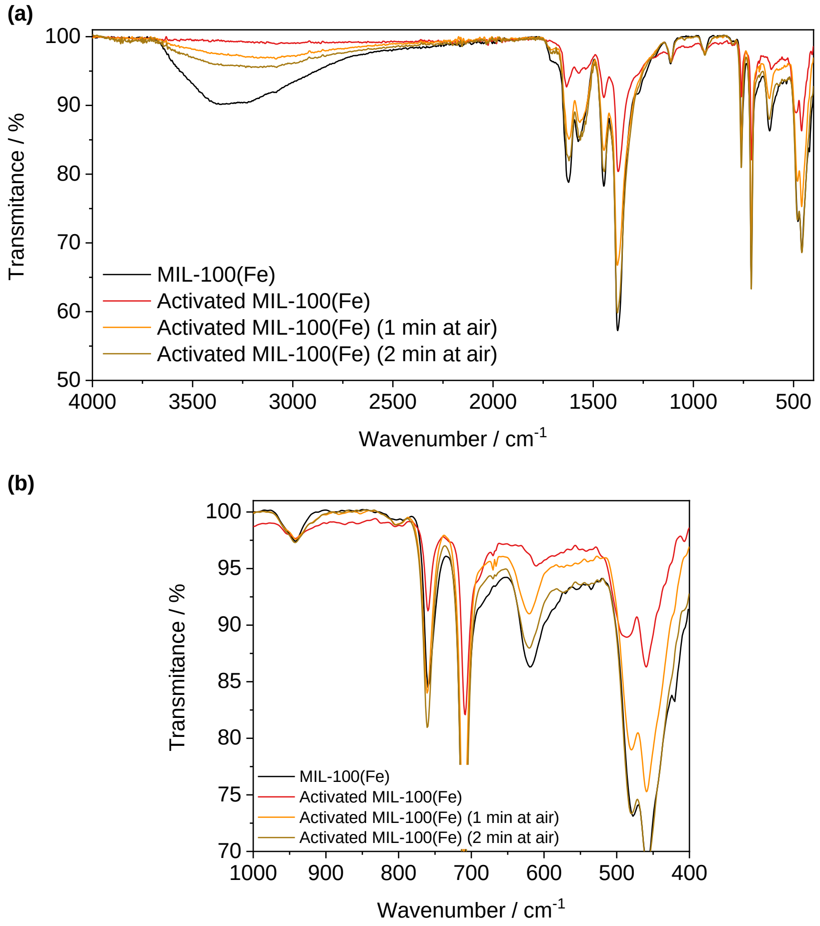

FIGURE S6: Infrared spectra comparison between (a) 4000-400 cm^{-1} and (b) 1000 to 400 cm^{-1} of MIL-100(Fe) before activation, activated with thermal treatment under vacuum (190 °C for 2 hours) and after air exposure.

X-Ray Powder Diffraction

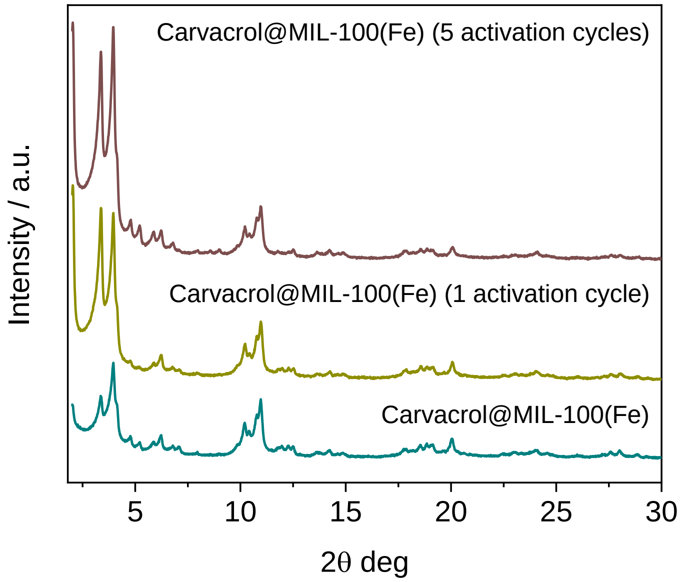

FIGURE S7: X-ray powder diffractograms of the encapsulation product before and after activation, and after 5 cycles of thermal treatment.

Mössbauer spectroscopy

Table S4: Estimated parameters from the Mössbauer spectra of the MIL-100(Fe) and carvacrol@MIL-100(Fe) samples taken at room temperature and 80 K.

Sample	T	IS (mm/s)	QS (mm/s)	I	Fe coordination number
MIL-100(Fe)	80 K	0.53	0.72	100%	Fe^{III} CN=6
Activated MIL-100(Fe)	80 K	0.52	0.96	100%	Fe^{III} CN=6 and CN=5
Carvacrol@MIL-100(Fe)	80 K	0.53	0.61	100%	Fe^{III} CN=6
Activated Carvacrol@MIL-100(Fe)	80 K	0.54	0.83	76%	Fe^{III} CN=6
		1.33	2.30	24%	Fe^{II} CN=6

IS average isomer shift relative to metallic α-Fe at 295 K; QS average quadrupole splitting; I estimated relative area. Estimated errors are ≤ 0.04 mm/s for IS and QS and ≤ 3% for I.

Electron paramagnetic resonance spectroscopy

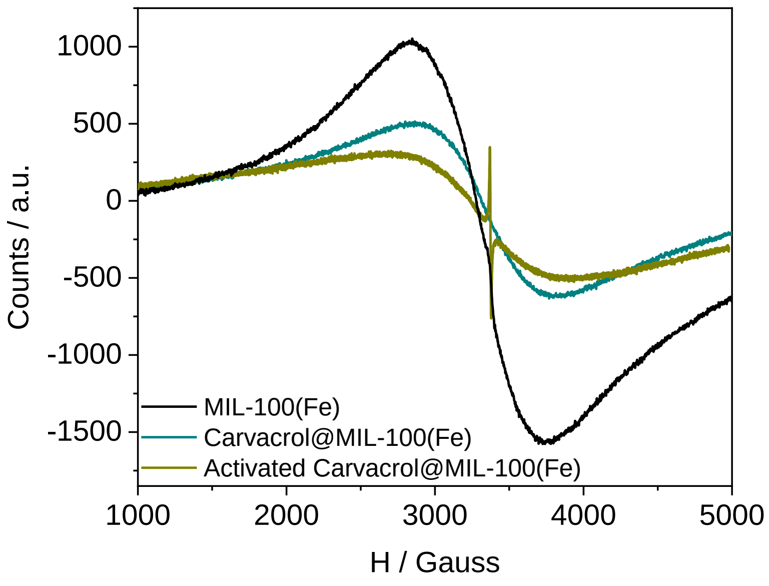

FIGURE S8: EPR spectra of Carvacrol@MIL-100(Fe) before and after activation, in comparison with pristine MIL-100(Fe). All measurements are performed at 300K.

Theoretical calculations

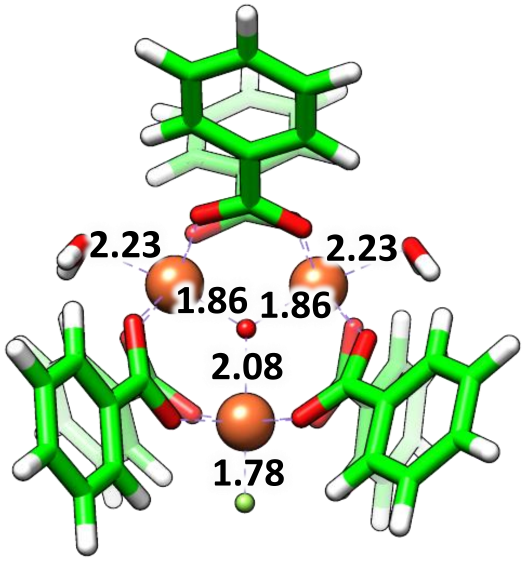

Figure S9. Minimum-energy structure calculated at the B3LYP-D3/6-31G(d,p) level of theory for the oxo-centered tri-metallic cluster of MIL-100(Fe) in a high-spin configuration for iron atoms. Relevant coordination distances are indicated in Å.

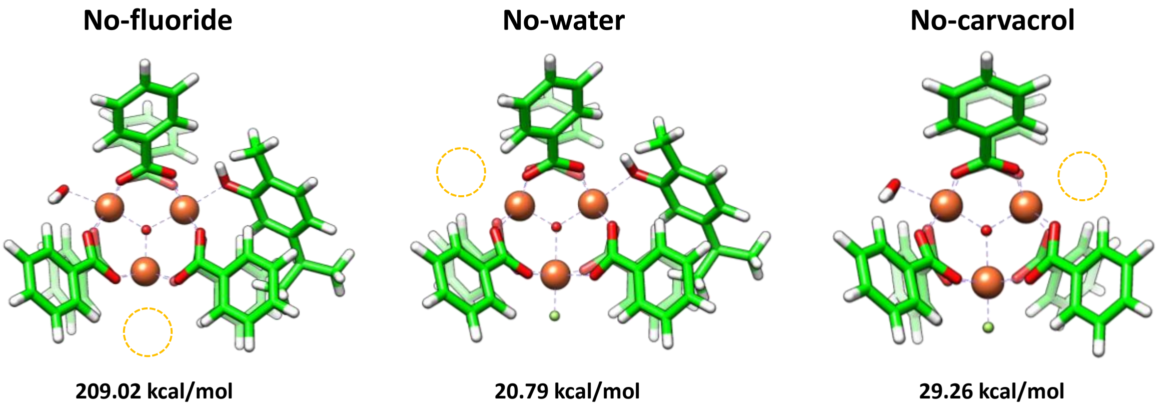

Figure S10. Energy required to detach a coordinated fluoride, water and carvacrol moiety from the tri-metallic cluster model of MIL-100(Fe) at the B3LYP-D3/6-31G(d,p) level.

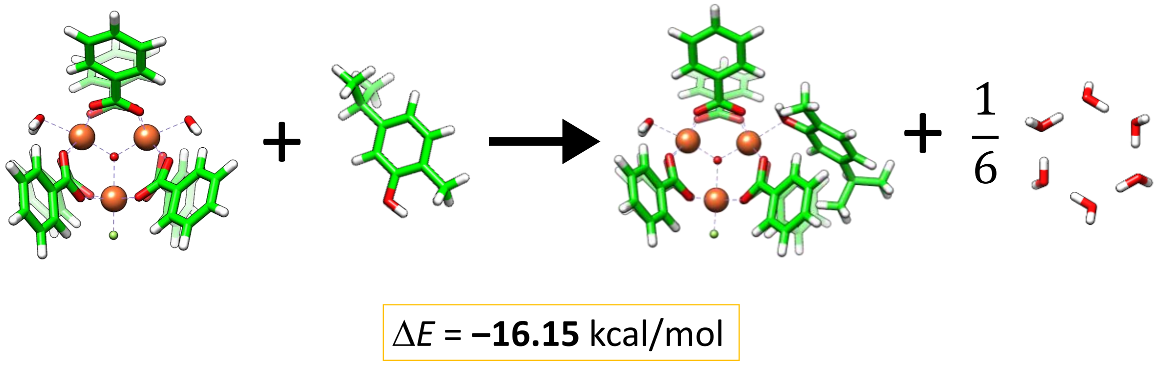

Figure S11. Energy difference for the substitution of a coordinated water molecule by carvacrol system calculated at the B3LYP-D3/6-31G(d,p) level.

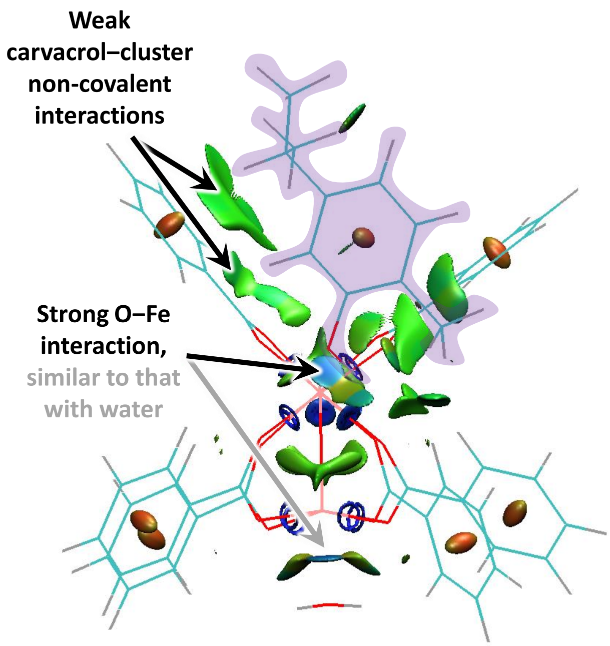

Figure S12. Non-covalent interaction (NCI) surfaces calculated for the tri-metallic cluster model upon carvacrol coordination. Green surfaces indicate weak non-covalent interactions whereas bluish surfaces represent stronger coordinative bonds.

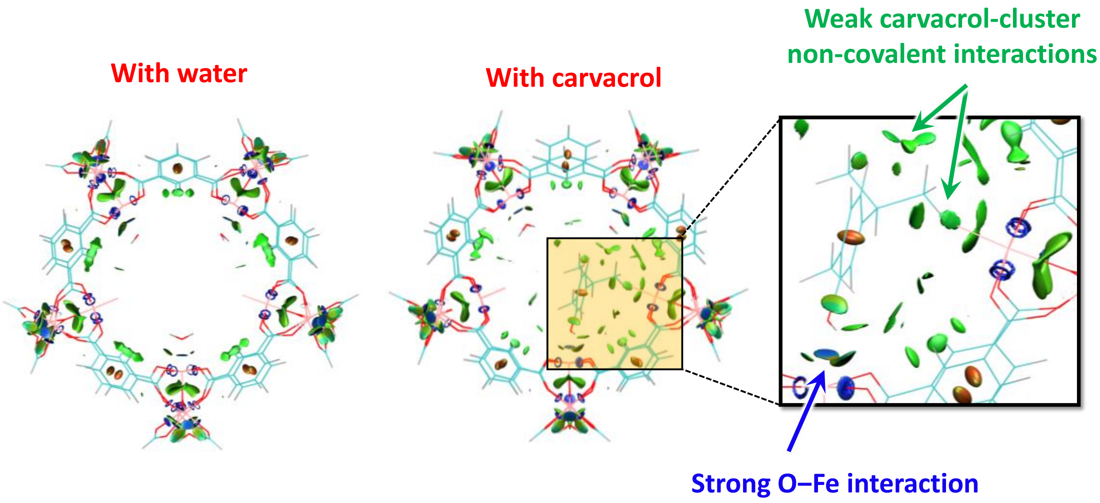

Figure S13. Non-covalent interaction (NCI) surfaces calculated for the pentagonal gate window model with coordination of water (left) and carvacrol (right).

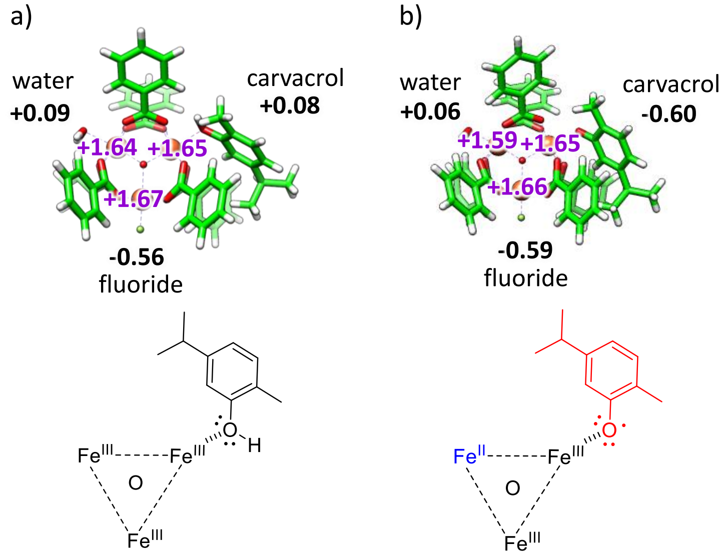

Figure S14. Natural population analysis (NPA) charges calculated at the B3LYP-D3/6-31G(d,p) level for the coordination of carvacrol molecule (a) and deprotonated carvacrol (b) in the tri-metallic cluster model of MIL-100(Fe). The corresponding chemical structure representation is drawn at the bottom.

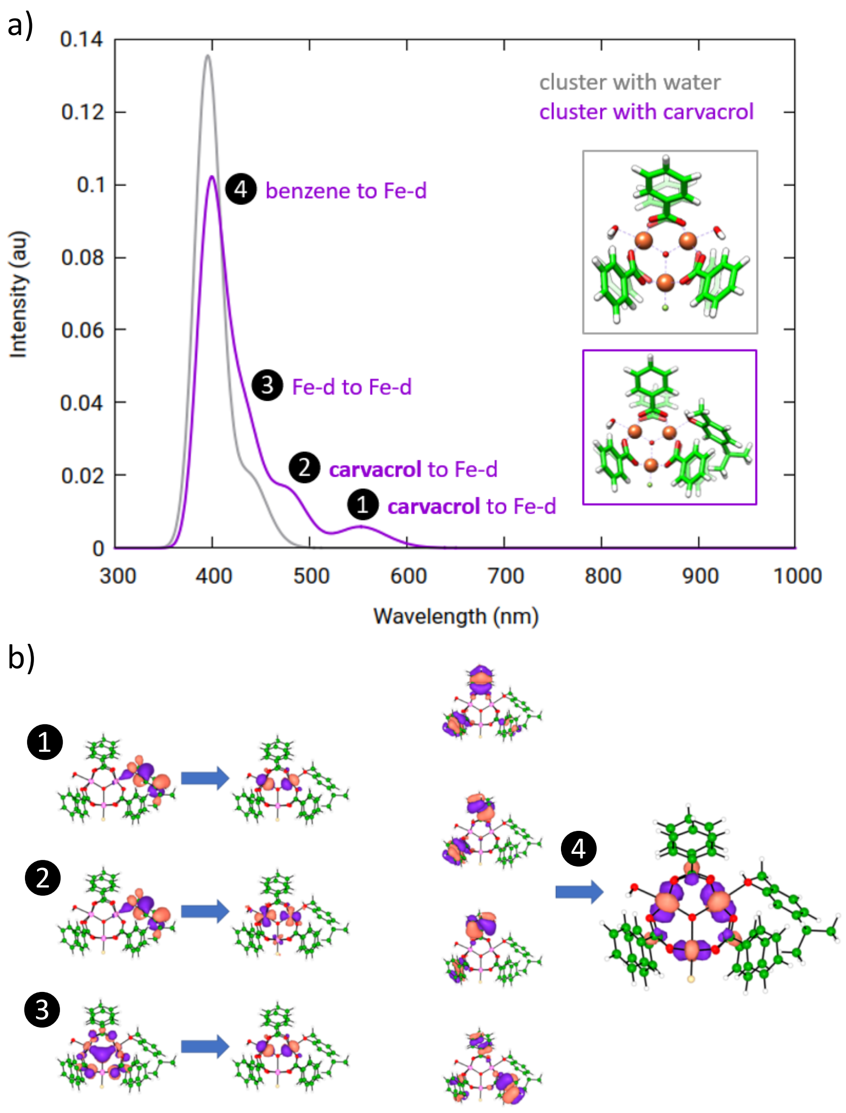

Figure S15. (a) Time-dependent DFT absorption spectra calculated for the tri-metallic cluster model with water (gray) and carvacrol (purple) coordination. (b) Monoelectronic excitations that describe the most relevant singlet excited states giving shape to the absorption spectrum of carvacrol@MIL-100(Fe) cluster system.

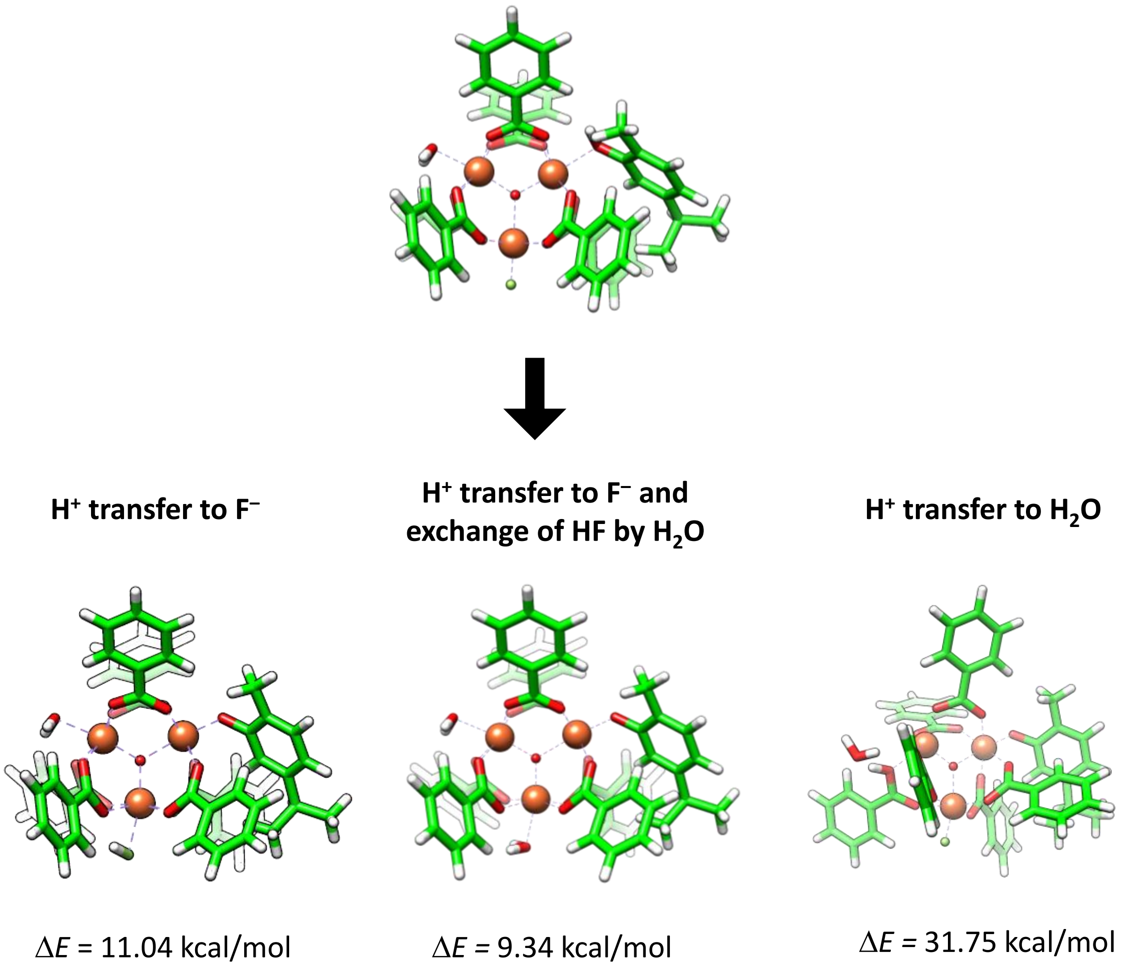

Figure S16. Energy penalty for the proton transfer from the hydroxy group of carvacrol to fluoride, to fluoride plus water exchange, or to water.

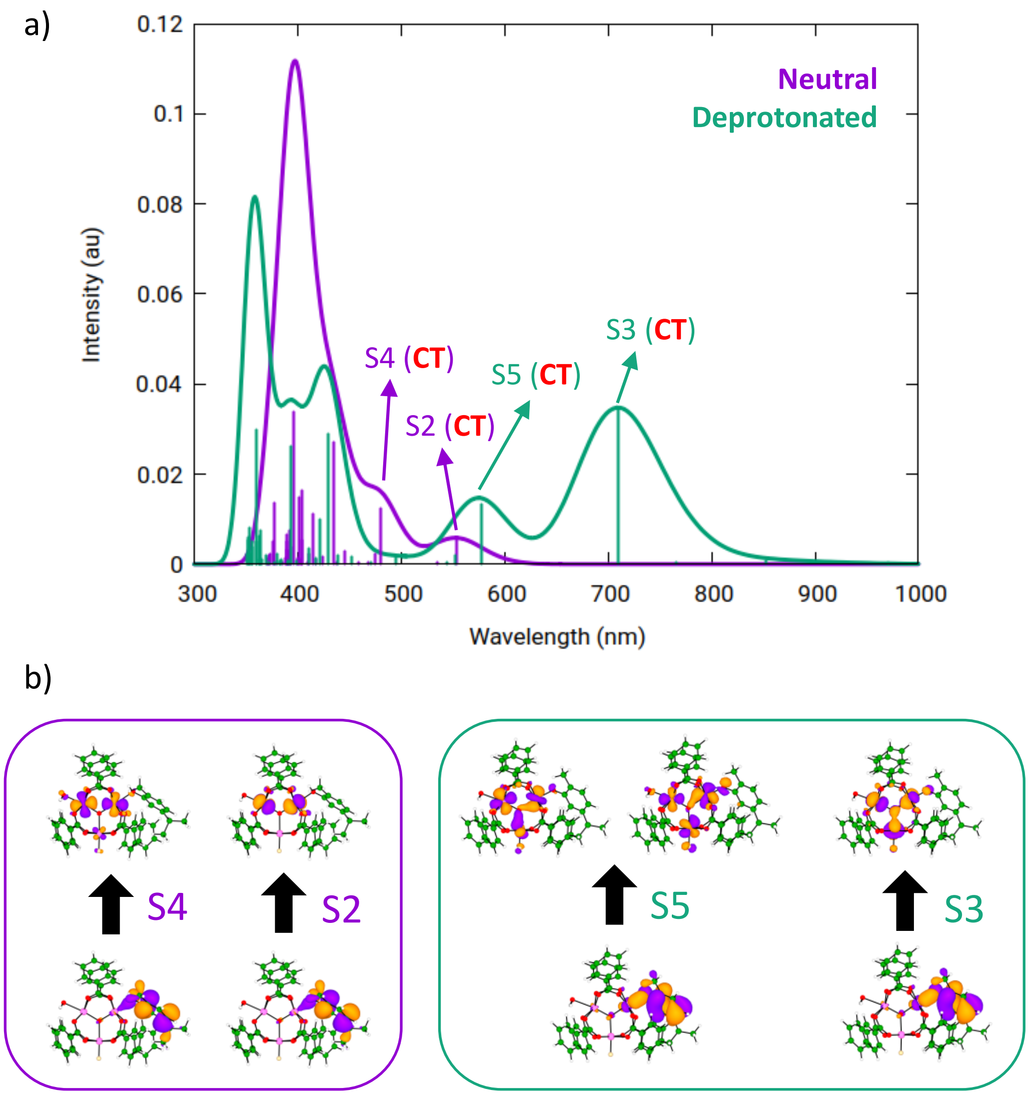

Figure S17. (a) Time-dependent DFT absorption spectra calculated for the carvacrol@MIL-100(Fe) cluster model with neutral protonated carvacrol (purple) and deprotonated carvacrol (green). b) Monoelectronic excitations that describe the most relevant singlet excited states giving shape to the absorption spectra for neutral (left) and deprotonated (right) carvacrol.
